# Congenital Heart Disease in a Paralympic Athlete With Klinefelter Syndrome

**DOI:** 10.1016/j.jaccas.2026.108748

**Published:** 2026-06-16

**Authors:** Francisco B. Alexandrino, Michael Villalonga, Nandini Mehra, Tara Karamlou, Joanna Ghobrial, Margaret Fuchs, Tamanna K. Singh

**Affiliations:** a Department of Internal Medicine, Cleveland Clinic, Cleveland, Ohio, USA; b Department of Cardiovascular Medicine, Heart, Vascular and Thoracic Institute, Cleveland Clinic, Cleveland, Ohio, USA; c Department of Cardiac Surgery, Akron Children's Hospital, Akron, Ohio, USA

**Keywords:** atrial septal defect, congenital heart disease, exercise capapulmonary/systemic flow ratiocity, Klinefelter syndrome, Paralympic

## Abstract

**Background:**

Sinus venosus atrial septal defects with partial anomalous pulmonary venous return (PAPVR) are rare congenital abnormalities.

**Case Summary:**

A 26-year-old competitive swimmer with Klinefelter syndrome was referred for evaluation after echocardiography revealed right-sided chamber dilation. Multimodality imaging confirmed sinus venosus atrial septal defects with PAPVR of the right upper and middle pulmonary veins draining into the superior vena cava (pulmonary/systemic flow ratio = 1.8). He underwent successful pericardial-patch closure and venous baffling to the left atrium. At 1 year, imaging showed intact repair and unobstructed venous flow. He resumed competitive swimming and remains asymptomatic while training for the 2028 Paralympic Games.

**Discussion:**

This case illustrates preserved exercise capacity and full postoperative recovery in an athlete with atrial septal defect-PAPVR.

**Take-Home Messages:**

Athletes with congenital heart disease and disability can safely pursue competitive sports. In atrial septal defect, preserved exercise capacity does not exclude hemodynamically significant congenital heart disease.

## Visual Summary

**Figure:**
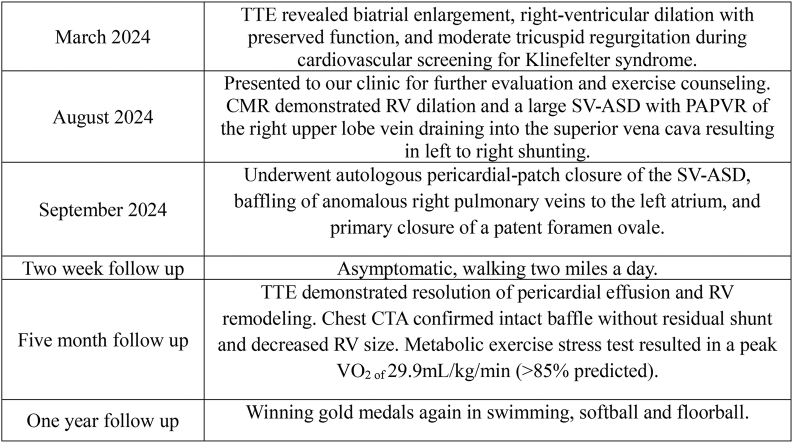

## History of Presentation

A 26-year-old competitive swimmer with Klinefelter syndrome presented in August 2024 for evaluation after an incidental finding of congenital heart disease. He described occasional “abnormal heart sensations” but denied dyspnea, palpitations, or exercise intolerance. One brief episode of chest discomfort had resolved with antacids and was attributed to gastroesophageal reflux disease.

**Figure 1:**
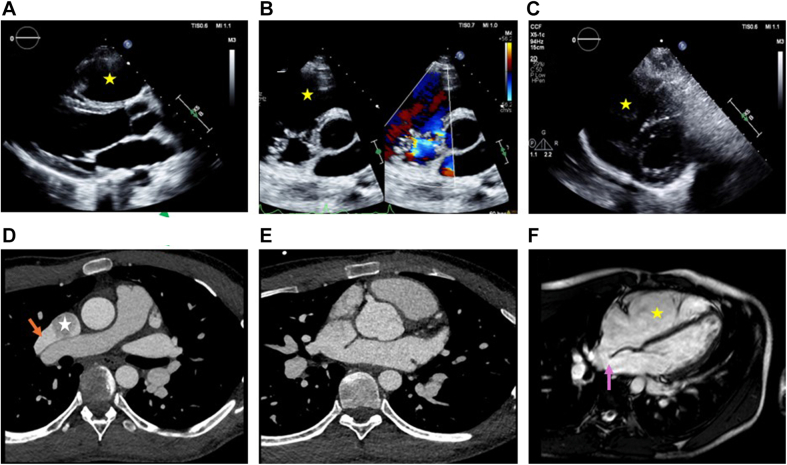
Preoperative Evaluation of Sinus Venosus Atrial Septal Defect and Partial Anomalous Pulmonary Venous Return (A) Parasternal long-axis view on transthoracic echocardiography showing right ventricular dilation (yellow star); (B) short-axis view on transthoracic echocardiography showing mild tricuspid regurgitation (red arrow) and dilated right ventricle (yellow star); (C) short-axis view on transthoracic echocardiography showing significant right ventricular dilation (yellow star); (D) right upper pulmonary vein (orange arrow) draining into the superior vena cava (white star); (E) right middle pulmonary vein draining into the right atrium at the level of the superior sinus venosus defect (F) cardiac magnetic resonance imaging showing the superior sinus venosus defect (purple arrow) and a dilated right ventricle (yellow star). CCF = coronary cameral fistula.

Two months earlier, during cardiovascular screening for Klinefelter syndrome, transthoracic echocardiography (TTE) revealed preserved left-ventricular systolic function, biatrial enlargement, right ventricular (RV) dilation with preserved function, and moderate tricuspid regurgitation (Figures 1A to 1C). He was referred for further evaluation and exercise counseling. Physical examination showed a tall, slender body habitus with normal heart sounds and no murmurs.

## Past Medical History

Klinefelter syndrome was diagnosed via amniocentesis and previously treated with testosterone. He also had a history of mild developmental delay.

## Differential Diagnosis

Differential diagnoses included acquired causes of right-sided chamber dilation such as pulmonary hypertension, significant tricuspid regurgitation, or right-sided cardiomyopathies including arrhythmogenic RV dysplasia. Congenital causes such as atrial septal defects, partial anomalous pulmonary venous connection, Ebstein anomaly, and other forms of anomalous pulmonary venous drainage should also be considered.

## Investigations

Cardiac magnetic resonance (CMR) demonstrated RV dilation and a large superior sinus venosus atrial septal defect (SV-ASD) with partial anomalous pulmonary venous return (PAPVR) of the right upper lobe vein draining into the superior vena cava (SVC), resulting in a left-to-right shunt (pulmonary/systemic flow ratio [Q_p_:Q_s_] = 1.8:1) (Figure 1F). A small transmural scar at the inferoapical septum suggested prior ischemic injury. Coronary computed tomography angiography excluded epicardial coronary disease and confirmed the SV-ASD (3.1 × 1.8 cm, area: 3.9 cm^2^) with anomalous drainage of the right upper and middle pulmonary veins and RV dilation (Figures 1D and 1E).

## Management

In September 2024, the patient underwent autologous pericardial-patch closure of the SV-ASD, baffling of anomalous right pulmonary veins to the left atrium, and primary closure of a patent foramen ovale. Intraoperative transesophageal echocardiography confirmed good repair with patent pulmonary venous drainage and no residual shunt.

The postoperative course was complicated by post-pericardiotomy syndrome, managed successfully with colchicine 0.6 mg twice daily and ibuprofen 600 mg 4 times daily.

## Outcome and Follow-Up

At 2-week follow-up, he was asymptomatic and walking 2 miles twice daily. He was eager to get back to swimming, but per the surgical guidelines for postoperative care, he was advised to avoid swimming for 6 weeks.

**Figure 2:**
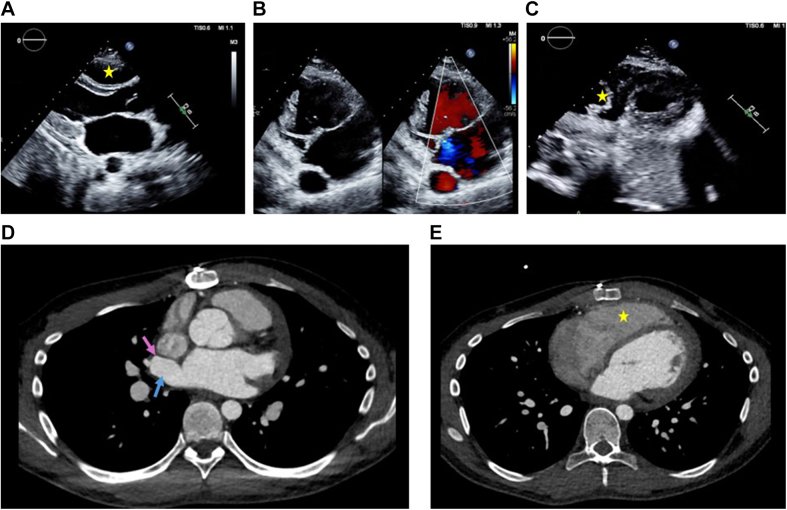
Postoperative Evaluation of Repaired Sinus Venosus Atrial Septal Defect and Partial Anomalous Pulmonary Venous Return (A) Parasternal long-axis view on transthoracic echocardiography showing decreased right ventricle size (yellow star); (B) right ventricle inflow view showing decreased tricuspid regurgitation; (C) short-axis view on transthoracic echocardiography showing decrease in right ventricle size (yellow star); (D) cardiac computed tomography patent right-sided pulmonary venous baffle (blue arrow) with closed superior sinus venosus defect (purple arrow); (E) cardiac computed tomography showing decrease in right ventricle size (yellow star).

At 5-month follow-up, TTE demonstrated resolution of the pericardial effusion, preserved left ventricular ejection fraction, decreased tricuspid regurgitation, and a significant decrease in RV dilation with an RV systolic pressure of 35 mm Hg. The intra-atrial venous baffle showed low-velocity, laminar flow with no evidence of obstruction, and a contrast study was positive for a small right-to-left shunt across the interatrial septum (Figures 2A to 2C). Chest computed tomography angiography confirmed an intact baffle without residual shunt, patent pulmonary veins with mild angulation and slight narrowing at the ostium of the vein draining the medial segment of the right middle lobe, and decreased RV size compared with prior imaging (Figures 2D and 2E).

A metabolic exercise stress test demonstrated a peak VO_2_ of 29.9 mL/kg/min (>85% predicted), peak heart rate of 174 beats/min, chronotropic response index of 0.83, peak blood pressure of 144/68 mm Hg, normal O_2_ saturation, and VE/VCO_2_ slope of 33 without desaturation episodes or significant electrocardiogram changes. We discussed a plan to resume normal vigorous physical activity with progressive return to competition levels. He completed a colchicine taper and was maintained on aspirin 81 mg daily, the only medication he currently takes.

At 1-year follow-up, he had resumed preoperative physical activities including team sports such as softball and floorball, besides swimming. He won gold medals in all 3. He reported completing 100 m of breaststroke in 1:07. For comparison, the men's Olympic record for the 100-m breaststroke is 57.13 seconds (Adam Peaty, Rio 2016) and the women's record is 1:04.82 (Tatjana Schoenmaker, Tokyo 2020). He continues to train with determination, aspiring to qualify for the 2028 Paralympic Games in Los Angeles.

## Discussion

We describe a young competitive swimmer with Klinefelter syndrome who was preparing for the Paralympic Games and was incidentally found to have a rare congenital anomaly that requires surgical correction. According to current guidelines, atrial septal defect (ASD) closure is indicated in the presence of symptoms, right atrial or ventricular enlargement, or a significant left-to-right shunt (Q_p_:Q_s_ ≥1.5:1). Our patient met 2 of these criteria, with marked RV dilation and a shunt fraction of 1.8, prompting surgical repair, a Class IIa guideline indication for surgery.^1^ This case's main teaching point is that preserved exercise capacity does not exclude hemodynamically significant congenital heart disease.

PAPVR is found in approximately 0.2% of the population,^2^ whereas ASDs occur in about 0.9%, both resulting in left-to-right shunting.^3^ Nearly half of patients with PAPVR have a concomitant ASD, and among these, up to 79% present with the superior sinus venosus type, in which the defect occurs posterior to the atrial septum at the junction with the SVC, such as in our patient. Chromosomal abnormalities account for 15%-20% of all congenital cardiovascular diseases.^4^ Klinefelter syndrome, first described in 1942, is the most common sex chromosome aneuploidy, affecting approximately 1 in 660 men.^5^ It is characterized by gynecomastia, small firm testes, azoospermia, elevated follicle-stimulating hormone, and hypogonadism, leading to infertility. Cardiovascular manifestations, though less recognized, include an increased prevalence of mitral valve prolapse and patent ductus arteriosus. To date, only one case has been reported of a patient with Klinefelter syndrome presenting with both ASD and PAPVR.^6^

Although preoperative cardiopulmonary exercise testing data are unavailable, our patient demonstrated preserved functional capacity as reflected by his ability to perform at a competitive swimming level. In this context, preserved exercise capacity refers to functional athletic performance rather than objective physiological capacity. Importantly, this occurred despite hemodynamically significant disease, as evidenced by a Q_p_/Q_s_ of 1.8 with associated RV dilation. In congenital heart disease, self-reported exercise capacity and quality-of-life measures often overestimate true functional capacity (peak VO_2_), likely because, unlike patients with acquired heart disease, these individuals have never experienced normal, unimpaired physical capacity, having been affected since birth. This is supported by a study of more than 500 patients with congenital heart disease, which demonstrates that self-estimated physical functioning poorly predicts objectively measured exercise capacity.^7^

From a mechanistic standpoint, preserved exercise capacity in the presence of an ASD may be explained by the ability to maintain systemic cardiac output during exertion. Stress CMR data suggest that with increasing heart rate, reduced diastolic filling time leads to a decrease in shunt volume per beat, resulting in a relatively stable shunt fraction despite rising total cardiac output. As a result, systemic output can increase appropriately even in the presence of left-to-right shunting. This physiological framework may contribute to our patient's preserved, and ultimately enhanced, performance despite a hemodynamically significant shunt.^8^

Following surgical repair, our patient demonstrated objective improvement in exercise capacity, reflected by faster competitive swimming times and a peak VO_2_ exceeding 85% of the predicted value. This functional gain is likely driven by favorable structural remodeling after defect closure, including regression of RV hypertrophy, reduction in RV dilation, and a concomitant decrease in pulmonary pressures. These findings are consistent with prior CMR data showing significant RV reverse remodeling at 12-month follow-up after ASD closure in patients with baseline RV dilation.^9^

Surgical outcomes for SV-ASD repair are consistently excellent and provide important context for this case. A contemporary meta-analysis of 40 studies including 1,320 patients demonstrated very low perioperative risk, with in-hospital mortality of 0.24% and 30-day mortality of 0.5%. Rates of residual defects and venous complications were similarly low, including reoperation in 1.36%, residual shunt in 1.34%, SVC obstruction in 1.76%, and pulmonary vein obstruction in 1.4%.^10^ These data underscore that our patient's favorable postoperative course and recovery were to be expected.

Consistent with this, current sports cardiology and adult congenital heart disease guidelines recommend a gradual return to exercise following successful ASD repair, with full return to competitive sports at approximately 12 months in patients who remain asymptomatic and without residual shunt, pulmonary hypertension, ventricular dysfunction, or clinically significant arrhythmias.^1^^,^^11^ Our patient's postoperative course and return to high-level athletic performance align well with these recommendations, further supporting the safety of resuming competitive activity in appropriately selected individuals.

This case shows that high-level athletic performance should not exclude the presence of hemodynamically significant congenital heart disease. Early identification and guideline-directed intervention can restore normal physiology and safely enable return to competitive sport.

## Conclusions

The patient's exceptional exercise capacity before the surgery can be explained by preserved systemic cardiac output and the absence of an increase in left-to-right shunting during exertion. His postoperative recovery further highlights that, in the absence of pulmonary hypertension, patients with ASD and PAPVR can achieve remarkable functional recovery following surgical repair. This case also highlights the importance of considering congenital heart anomalies in individuals with Klinefelter syndrome and emphasizes that excellent exercise tolerance does not exclude significant structural disease. Beyond its clinical implications, this case reinforces our collective goal of empowering patients with adult congenital heart disease to safely engage in sports and pursue active, fulfilling lives. Our patient remains asymptomatic and continues to train toward participation in the 2028 Paralympic Games.

## Funding Support and Author Disclosures

The authors have reported that they have no relationships relevant to the contents of this paper to disclose.

**Take-Home Messages**

• Athletes with congenital heart disease and disability can safely pursue competitive sports.
• In atrial septal defect, preserved exercise capacity does not exclude hemodynamically significant congenital heart disease.

**Visual Summary:** A Paralympic Athlete With Klinefelter Syndrome Underwent Successful epair of Sinus Venosus ASD With PAPVR and Returned to Elite Competition With Preserved Cardiopulmonary Performance

March 2024	TTE revealed biatrial enlargement, right ventricular dilation with preserved function, and moderate tricuspid regurgitation during cardiovascular screening for Klinefelter syndrome.
August 2024	Presented to our clinic for further evaluation and exercise counseling. CMR demonstrated RV dilation and a large SV-ASD with PAPVR of the right upper lobe vein draining into the SVC, resulting in left-to-right shunting.
September 2024	Underwent autologous pericardial-patch closure of the SV-ASD, baffling of anomalous right pulmonary veins to the left atrium, and primary closure of a patent foramen ovale.
2-wk follow-up	Asymptomatic, walking 2 miles a day.
5-mo follow-up	TTE demonstrated resolution of pericardial effusion and RV remodeling. Chest CTA confirmed an intact baffle without residual shunt and decreased RV size. Metabolic exercise stress test resulted in a peak VO_2 of_ 29.9 mL/kg/min (>85% predicted).
1-y follow-up	Winning gold medals again in swimming, softball, and floorball.

CMR = cardiac magnetic resonance imaging; CTA = computed tomography angiography; PAPVR = partial anomalous pulmonary venous return; RV = right ventricle; SV-ASD = sinus venosus atrial septal defect; SVC = superior vena cava; TTE = transthoracic echocardiography; VO_2_ = oxygen consumption.
